# Arterial and venous thrombosis in coronavirus 2019 disease (Covid-19): relationship with mortality

**DOI:** 10.1007/s11739-020-02621-8

**Published:** 2021-07-04

**Authors:** Francesco Violi, Giancarlo Ceccarelli, Roberto Cangemi, Francesco Cipollone, Damiano D’Ardes, Alessandra Oliva, Matteo Pirro, Monica Rocco, Francesco Alessandri, Gabriella D’Ettorre, Miriam Lichtner, Pasquale Pignatelli, Domenico Ferro, Franco Ruberto, Gregory Y. H. Lip, Francesco Pugliese, Claudio Maria Mastroianni, the Intensive Care, Infectious Diseases COVID-19 Study Group of Sapienza University, Francesco Pugliese, Francesco Alessandri, Franco Ruberto, Alida Albante, Daniela Auricchio, Francesco De Lazzaro, Daniela M. De Lauri, Carmela Di Santo, Stefano Ianni, Eugenia Magnanimi, Fabiola Ratini, Anna Sabani, Luca Titi, Paola Vaccaro, Giovanni Giordano, Chiara Manganelli, Massimo Mancone, Katia Bruno, Paola Celli, Stella Consolo, Claudia Croce, Lorena Giannetti, Sabina Martelli, Teresa Messina, Elisa Pattelli, Serena Perrella, Monica Portieri, Claudia Ricci, Nicole Almenrader, Roberto Arzilla, Emilia Delia, Claudio Di Giovanni, Amalia Laderchi, Carlotta Macrì, Maurizio Marandola, Giada Nardecchia, Massimo Pacilli, Francesca Pacini, Fabio Araimo Morselli, Carmela Imperiale, Paolo Tordiglione, Claudio Maria Mastroianni, Maria Rosa Ciardi, Camilla Ajassa, Gabriella D’Ettorre, Miriam Lichtner, Claudia D’Agostino, Gianluca Russo, Vito Trinchieri, Paola Guariglia, Laura Antonelli, Alessandra Oliva, Rosaria Maria Cuomo, Martina Carnevalini, Cristina Mastropietro, Giancarlo Iaiani, Ivano Mezzaroma, Mario Falciano, Giancarlo Ceccarelli, Andrea Brogi, Luigi Celani, Nelson Eugenio Cavallari, Marco Rivano Capparuccia, Anna Paola Massetti, Caterina Fimiani, Marta Santori, Alessandro Bianchi, Cristiana Franchi, Maurizio De Angelis, Silvia Sereno, Caterina Furlan, Giuseppe De Sanctis, Francesca Paoletti, Patrizia Pasculli, Francesco Cogliati Dezza, Paolo Vassalini, Francesca Cancelli, Gabriella De Girolamo, Giulia Savelloni, Serena Valeri, Guido Siccardi, Federica Alessi, Gregorio Recchia, Marco Ridolfi, Francesco Eugenio Romani, Raissa Aronica, Valeria Filippi, Mauro Vera, Lorenzo Volpicelli, Matteo Candy, Rugova Alban, Silvia Di Bari, Francesca Gavaruzzi, Elena Casali, Maria Serena Carli, Antonella Maria Zingaropoli, Valentina Perri, Massimiliano De Angelis, Letizia Santinelli, Claudia Pinacchio, Parni Nijhawan, Claudia Maria Miele, Pietro Giuseppe Innocenti, Fabio Mengoni

**Affiliations:** 1 I Clinica Medica, Department of Clinical Internal, Anaesthesiologic and Cardiovascular Sciences, Sapienza University of Rome, Viale del Policlinico 155, 00161 Roma, Italy; 2 Mediterranea Cardiocentro, Naples, Italy; 3 Department of Public Health and Infectious Diseases, Sapienza University of Rome, Rome, Italy; 4 Department of Translational and Precision Medicine, Sapienza University of Rome, Rome, Italy; 5 Clinica Medica, Department of Medicine and Aging, “G. D’Annunzio, University of Chieti-Pescara, Chieti, Italy; 6 Unit of Internal Medicine, Department of Medicine, University of Perugia, Perugia, Italy; 7 Dipartimento Emergenza E Accettazione, Ospedale Universitario Sant’Andrea, Rome, Italy; 8 Department of General Surgery Paride Stefanini, Sapienza University of Rome, Rome, Italy; 9 Liverpool Centre for Cardiovascular Science, University of Liverpool and Liverpool Heart & Chest Hospital, Liverpool, UK; 10 Aalborg Thrombosis Research Unit, Department of Clinical Medicine, Aalborg University, Aalborg, Denmark

**Keywords:** Covid-19, SARS-cov-2, Thrombosis, Mortality

## Abstract

**Background:**

Patients with coronavirus disease 2019 (Covid-19) may experience venous thrombosis while data regarding arterial thrombosis are sparse.

**Methods:**

Prospective multicenter study in 5 hospitals including 373 patients with Covid-19-related pneumonia. Demographic data, laboratory findings including coagulation tests and comorbidities were reported. During the follow-up any arterial or venous thrombotic events and death were registered.

**Results:**

Among 373 patients, 75 (20%) had a thrombotic event and 75 (20%) died. Thrombotic events included 41 venous thromboembolism and 34 arterial thrombosis. Age, cardiovascular disease, intensive care unit treatment, white blood cells, D-dimer, albumin and troponin blood levels were associated with thrombotic events. In a multivariable regression logistic model, intensive care unit treatment (Odds Ratio [OR]: 6.0; 95% Confidence Interval [CI] 2.8–12.6; *p* < 0.001); coronary artery disease (OR: 2.4; 95% CI 1.4–5.0; *p* = 0.022); and albumin levels (OR: 0.49; 95% CI 0.28–0.87; *p* = 0.014) were associated with ischemic events. Age, sex, chronic obstructive pulmonary disease, diabetes, heart failure, coronary heart disease, intensive care unit treatment, in-hospital thrombotic events, D-dimer, C-reactive protein, troponin, and albumin levels were associated with mortality. A multivariable Cox regression analysis showed that in-hospital thrombotic events (hazard ratio [HR]: 2.72; 95% CI 1.59–4.65; *p* < 0.001), age (HR: 1.035; 95% CI 1.014–1.057; *p* = 0.001), and albumin (HR: 0.447; 95% CI 0.277–0.723; *p* = 0.001) predicted morality.

**Conclusions:**

Covid-19 patients experience an equipollent rate of venous and arterial thrombotic events, that are associated with poor survival. Early identification and appropriate treatment of Covid-19 patients at risk of thrombosis may improve prognosis.

## Introduction

Covid-19 is a serious pandemic characterized by severe acute respiratory disease needing mechanical ventilation and intensive care unit (ICU) treatment.

Among the factors predisposing to poor survival, thrombotic complications have been suggested to have an important role. Accordingly, clinical studies showed a high prevalence of venous thromboembolism in the Covid-19 patient population with a variable incidence, ranging from 7 to 31% [1]; a small series of autoptic studies demonstrated a relationship between thrombosis in the circulation lung and poor survival [2]. Conversely, the incidence of arterial thrombosis has been studied in small series and appears to be much lower than venous thromboembolism; in a recent review arterial thrombosis was reported to range from 2.8 to 3.8% [3]. While there are consistent data on the association between hypercoagulability and mortality [4], the impact of venous and arterial thrombosis on survival is less clear. Thus, our aim was to evaluate the occurrence of thrombotic events in the artery and venous circulation, and its relationship with mortality in patients recruited by 5 Italian hospital centers dedicated to the management of severe acute respiratory syndrome coronavirus 2019 (SARS-Cov-2).

## Methods

### Study design and population

This is a multicentre observational cohort study performed at University hospitals located in Rome (2 centres), Latina, Perugia and Chieti (all in Italy).

We included consecutive adult patients (age ≥ 18 years) with confirmed Covid-19-related pneumonia, with or without mechanical ventilation, hospitalized from 1 to 31st March 2020 and admitted to infectious disease wards or ICU. Covid-19 was diagnosed on the basis of the WHO interim guidance [5]. A confirmed case was defined as a person with laboratory confirmation of Covid-19 infection, irrespective of clinical signs and symptoms. Oropharyngeal and nasopharyngeal swabs for laboratory diagnosis of Covid-19 were performed in duplicate: SARS- Cov2 *E and S gene* were detected by a reverse transcriptase-polymerase chain reaction (RT-PCR).

High-resolution computed tomography (CT) scan was used to identify lung involvement according to the Official diagnosis and treatment protocol (6th edition) declared by the National Health Commission of China. Typical CT findings of SARS-CoV2 related pneumonia were considered: consolidation, ground-glass opacities, crazy paving and/or reticular pattern [6]. Radiologic abnormalities were reviewed by attending physicians in respiratory medicine who extracted the data. Major disagreement between two reviewers was resolved by consultation with a third reviewer. Ethical approval for this study was obtained from the Ethics Committee of “Azienda Ospedaliero Universitaria Policlinico Umberto I” (*approval number*/ID Prot. 109/2020).

#### Baseline assessment

Demographic, clinical, laboratory and radiological results were extracted from electronic medical records of patients enrolled. Cancer history was reported on the basis of the history-taking or from medical records. Laboratory assessments consisted of routine blood and chemical analysis included coagulation tests (D-dimer, prothrombin time (PT) and activated partial thromboplastin time (aPTT) and fibrinogen), high-sensitivity C-reactive protein (hs-CRP), procalcitonin and High-Sensitivity Cardiac Troponin T (hs-cTnT). Prevalence of diabetes mellitus, hypertension, coronary artery disease (CAD), chronic kidney disease, obesity were recorded, defined as previously described [7]. *Assessment of in-hospital thrombotic events.*

Patients were followed-up until discharge or in-hospital death. We registered the occurrence of thrombotic events including ischemic/embolic events that were categorized as follows: pulmonary embolism (PE), detected by lung CT scan [8]; *deep vein thrombosis (DVT), assessed by ultrasonography or by CT scan; superficial vein thromboses (SVT), assessed by ultrasonography in symptomatic patients;* acute myocardial infarction, diagnosed on the basis of the diagnostic electrocardiographic findings associated with elevation of serum markers *of m*yocardial necrosis [9]; acute ischemic strokes, identified by observing the onset of new focal neurological signs and symptoms and confirmed with MRI or CT imaging [10]; acute limb ischemia diagnosed according to guidelines [11]. *Inferior leg ultrasonography was performed in patients with signs and/or symptoms for inferior leg DVT and routinely performed in patients with PE. Uncommon vein thrombosis in symptomatic patients (i.e. gonadic vein thrombosis) was detected by CT-scan.*

#### Statistical analysis

Categorical variables are reported as counts and percentages and continuous variables as mean ± standard deviation (SD), or medians and interquartile ranges (IQRs). Differences between percentages were assessed by chi-square or Fisher exact tests. All continuous variables were tested for normality with the Shapiro–Wilk test. Student unpaired t-tests were used for normally distributed continuous variables. Appropriate nonparametric tests (Mann–Whitney and Spearman rank correlation tests) were used for the other variables. The bivariate and multivariate effects of prognostic factors on thrombotic events were assessed by means of logistic regression models. Wald confidence intervals and tests for odds ratios (OR) and adjusted OR were computed on the basis of the estimated standard errors. Survival curves were estimated using the Kaplan–Meier product-limit estimator and compared using the log-rank test. Cox proportional hazards analysis was used to calculate the adjusted hazard ratios (HR) and 95% confidence interval (CI) for each clinical variable. Only p values < 0.05 were considered statistically significant. All tests were 2-tailed, and analyses were performed using computer software packages (IBM SPSS Statistics 25).

## Results

### Thrombotic events in Covid-19

Three-hundred seventy-three patients were hospitalized for a median length of stay of 18 days (IQR: 12–27), 306 (82%) have been admitted to infectious disease wards and 67 (18%) in ICU. Baseline clinical and laboratory characteristics, as well as in-hospital treatments at admission, are reported in Table 1. During the in-hospital stay, 75 (20%) patients experienced thrombotic events: 41 (54%) were in the venous circulation (7 superficial venous thrombophlebitis, 1 gonadic venous thrombosis, 15 deep venous thrombosis, 18 pulmonary embolism) and 34 (46%) were in the artery circulation (9 acute limb ischemia, 15 acute myocardial ischemia and 10 TIA/strokes).

**Table 1:** Clinical and laboratory characteristics of study patients, according to thrombotic events

	All patients	TE-free patients	Patients with TE	P value
N	373	298	75	
Age	67.4 ± 16.8	66.3 ± 17.0	71.5 ± 15.5	0.012
Male sex	61%	60%	67%	0.295
ICU admission	18%	12%	41%	< 0.001
Hypertension	53%	51%	61%	0.117
Diabetes	17%	17%	17%	0.977
Smoking habit	15%	14%	16%	0.857
COPD	12%	12%	13%	0.761
CAD	15%	13%	25%	0.015
Heart failure	19%	18%	23%	0.320
Atrial fibrillation	13%	12%	16%	0.448
**Cancer history**	**11%**	**11%**	**13%**	**0.707**
ACE-inhibitors	18%	16%	25%	0.193
ARBs	13%	14%	11%	0.677
Aspirin	15%	13%	21%	0.207
Statins	15%	13%	22%	0.116
**Heparins**	**81%**	**80%**	**85%**	**0.326**
**PaO**_**2**_**/FiO**_**2**_	**314 [252–362]**	**326 [281–376]**	**256 [158–310]**	** < 0.001**
WBC (× 1000/mm3)	7.24 ± 3.86	6.78 ± 3.76	8.51 ± 4.15	0.010
PLT (× 1000/mm3)	210 ± 85	207 ± 76	218 ± 114	0.388
Creatinine (mg/dl)	1.01 ± 0.45	0.99 ± 0.49	1.07 ± 0.31	0.334
AST (U/L)	27 [21–42]	26 [21–40]	31 [24–49]	0.117
ALT (U/L)	23 [16–38]	22 [16–37]	25 [18–42]	0.704
hs-CRP (mg/L)	52 [20–131]	48 [19–120]	82 [21–170]	0.081
D-dimer (ng/ml)	1189 [640–2765]	1140 [610–2016]	2390 [1275–4800]	< 0.001
hs-cTnT (ng/ml)	0.010 [0.004–0.031]	0.08 [0.004–0.019]	0.023 [0.005–0.065]	0.002
Albumin (g/dL)	3.39 ± 0.55	3.46 ± 0.53	3.16 ± 0.56	< 0.001

TE; thrombotic events; ICU: intensive care unit; WBC: white blood cells, PLT: platelets; hs-CRP: high-sensitivity C reactive protein; hs-cTnT: high sensitivity cardiac troponin T; ACE: angiotensin-converting enzyme inhibitors; ARBs: angiotensin receptor blockers; CAD: coronary heart disease; COPD: chronic obstructive pulmonary disease

Data are expressed as mean ± standard deviation or median [interquartile range]

Compared to patients who did not experience an in-hospital thrombotic event, patients with thrombotic events were older (71.5 ± 15.5 vs. 66.3 ± 17.0; *p* = 0.012), were more likely to have a history of CAD (25% vs. 13%; *p* = 0.015), and to have been admitted to ICU (41% vs. 12% *p* < 0.001). Moreover, PaO_2_/FiO_2_ ratio at hospital admission were lower in patients who developed thrombotic events. Among the laboratory variables, baseline values of white blood cells, serum D-dimer, high sensitivity cardiac troponin T (hs-cTnT), were higher, and albumin lower, in patients who developed thrombotic events (Table 1).

In a multivariable regression logistic model, ICU admission (OR. 6.0; 95% CI 2.8–12.6; *p* < 0.001), CAD (OR: 2.4; 95% CI 1.4–5.0; *p* = 0.022), and low albumin levels (OR: 0.49; 95% CI 0.28–0.87; *p* = 0.014) were independently associated with thrombotic events.

When separately analyzed, no significant differences in comorbidities were found between patients who experienced in-hospital arterial or venous thrombotic events. However, patients with arterial events showed to be older and higher baseline hs-cTnT, while patients with venous event showed lower PaO_2_/FiO_2_ (Table 2).

**Table 2:** Clinical and laboratory characteristics of study patients, according to arterial of venous thrombotic events

	Arterial events	Venous events	P value
N	34	42	
Age	75.8 ± 13.0	69.3 ± 12.2	0.028
Male sex	56%	76%	0.088
ICU admission	30%	51%	0.064
Hypertension	56%	66%	0.470
Diabetes	18%	17%	1.000
Smoking habit	15%	17%	1.000
COPD	9%	17%	0.480
CAD	26%	21%	0.782
Heart failure	29%	16%	0.256
Atrial fibrillation	21%	11%	0.329
**Cancer history**	13%	14%	1.000
ACE-inhibitors	27%	22%	0.787
ARBs	9%	11%	0.722
Aspirin	23%	19%	0.780
Statins	26%	19%	0.582
Heparins	82%	87%	0.532
PaO_2_/FiO_2_	290 [257–234]	171 [130–280]	< 0.001
WBC (× 1000/mm3)	7.47 ± 3.39	9.14 ± 4.145	0.194
PLT (× 1000/mm3)	212 ± 97	227 ± 130	0.609
hs-CRP (mg/L)	91 [36–162]	45 [19–178]	0.517
D-dimer (ng/ml)	2486 [882–4800]	2360 [1320–4725]	0.707
hs-cTnT (ng/ml)	0.038 [0.018–0.079]	0.008 [0.003–0.048]	0.012
Albumin (g/dL)	3.13 ± 0.57	3.19 ± 0.55	0.569

ICU: intensive care unit; WBC: white blood cells, PLT: platelets; hs-CRP: high-sensitivity C reactive protein; hs-cTnT: high sensitivity cardiac troponin T; ACE: angiotensin-converting enzyme inhibitors; ARBs: angiotensin receptor blockers; CAD: coronary heart disease; COPD: chronic obstructive pulmonary disease

Data are expressed as mean ± standard deviation or median [interquartile range]

### Mortality in Covid-19

**Fig. 1:**
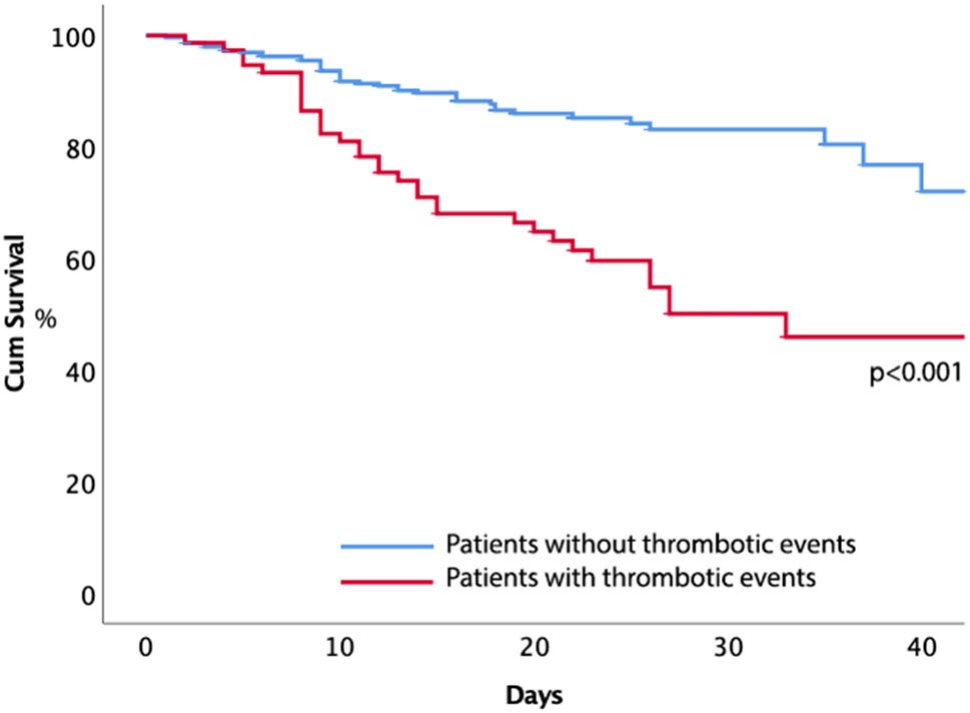
Kaplan–Meier estimates of time to mortality in patients with or without thrombotic events during the in-hospital stay

During the in-hospital follow-up, 75 (20%) patients died. Clinical and laboratory characteristics of patients according to mortality are represented in Table 3. Patients who died were older, more likely male and with a greater prevalence of COPD, diabetes, heart failure, coronary artery disease, ICU admission and intra-hospital thrombotic events than patients who survived. Among the laboratory variables, baseline D-dimer, hs-CRP, hs-cTnT, and albumin levels were associated with mortality. Kaplan-Meier analysis showed that patients with thrombotic events had a higher probability of in-hospital mortality compared to thrombotic events-free patients (44% vs. 14%; *p* < 0.001) (Fig. 1). A multivariable COX regression analysis showed that in-hospital thrombotic events (HR: 2.481; 95% CI 1.336–4.609; *p* = 0.004), age (HR: 1.048; 95% CI 1.022–1.075; *p* < 0.001), baseline low albumin levels (HR: 0.447; 95% CI 0.277–0.723; *p* = 0.001) and low PaO_2_/FiO_2_ ratio (HR: 0.996; 95% CI0.993–0.999; *p* = 0.022) predicted mortality, after adjusting for sex, ICU admission, diabetes, CAD, heart failure and COPD.

**Table 3:** Clinical and laboratory characteristics of study patients, according to mortality

	Survivors	Non-survivors	p
N	298	75	
Age	65.5 ± 17.0	75.3 ± 13.9	< 0.001
Male sex	59%	72%	0.035
ICU admission	11%	44%	< 0.001
Hypertension	51%	61%	0.139
Diabetes	15%	25%	0.049
Smoking habit	14%	18%	0.510
COPD	10%	23%	0.004
Cardiovascular disease	13%	22%	0.070
Heart failure	15%	36%	< 0.001
Atrial fibrillation	11%	19%	0.134
**Cancer history**	11%	15%	0.320
ACE-inhibitors	17%	21%	0.647
ARBs	14%	9%	0.480
Aspirin	12%	26%	0.040
Statins	12%	26%	0.038
**Heparins**	80%	84%	0.513
**P/F ratio**	328 [280–376]	256 [168–309]	< 0.001
WBC (× 1000/mm3)	7.00 ± 3.88	8.03 ± 3.77	0.141
PLT (× 1000/mm3)	211 ± 75	204 ± 119	0.662
Creatinine (mg/dl)	0.96 ± 0.40	1.19 ± 0.58	0.023
AST (U/L)	27 [21–40]	27 [19–50]	0.852
ALT (U/L)	22 [16–34]	23 [14–49]	0.501
hs-CRP (mg/L)*	45 [16–115]	92[43–208]	< 0.001
D-dimer (ng/ml)*	1160 [618–2230]	1849 [799–4632]	0.025
Troponin (ng/ml)	0.008 [0.004–0.019]	0.028 [0.010–0.064]	< 0.001
Albumin (g/L)	3.49 ± 0.53	3.03 ± 0.51	< 0.001
Intra-hospital TE	14%	44%	< 0.001

TE: thrombotic events; ICU: intensive care unit; WBC: white blood cells, PLT: platelets; hs-CRP: high-sensitivity C reactive protein; hs-cTnT: high sensitivity cardiac troponin T; ACE: angiotensin-converting enzyme inhibitors; ARBs: angiotensin receptor blockers; COPD: chronic obstructive pulmonary disease

Data are expressed as mean ± standard deviation or median [interquartile range]

## Discussion

The study shows that patients hospitalized for Covid-19 experience both venous and arterial ischemic events, that are a warning sign of poor survival.

Compared to previous reports showing that Covid-19 is prevalently associated to venous thromboembolism [3], the present study shows, conversely, that SARS-Cov-2 is complicated by an equipollent rate of venous and arterial thrombosis; thus among 75 patients with ischemic events 34 (46%) experienced arterial thrombosis, which occurred in the coronary, cerebral and peripheral circulation. The incidence of thrombosis was even higher than that reported in community-acquired pneumonia, which is essentially associated with arterial thrombosis [12–14], and was detectable infrequent sites of thrombosis such as, for example, superficial or gonadic venous thrombosis or acute leg ischemia.

The mechanisms accounting for thrombosis -related vascular complications may be dependent on the severe inflammatory response which accompanies the disease; this may activate platelets and clotting system via oxidative stress or over-production of cytokines with pro-thrombotic properties [15–18]; in accordance with this, we found an independent association between elevated D-dimer and thrombotic complications. Hypoalbuminemia may be another precipitating factor as it is associated with an increased risk of arterial and venous thrombosis [19, 20]. The present study supports and extends the results of a previous study from our group including a small group of Covid-19 population and reporting a close association between hypoalbuminemia and thrombosis [21]. Hypoalbuminemia is a feature of acute and chronic inflammation, as depicted by the inverse association with hs-CRP, and might favor thrombosis at the site of vascular lesion as albumin encompasses anticoagulant [19] and antiplatelet properties [22, 23]. However, we cannot exclude that other mechanisms such as increased albuminuria may also be implicated [19]; conversely, hypoalbuminemia as a reflection of concomitant acute liver failure can be reasonably excluded by the present and previous studies [4, 24].

The present study reinforces and extends the results of an autoptic study indicating that thrombosis predisposes to poor survival; thus, on multivariable analysis, intra-hospital venous and arterial thrombotic events along with age and albumin were factors associated with death. The novelty of the study is, as depicted by Kaplan-Meyer survival analysis, in the divergence of the curves within the first 10 days from hospitalization, suggesting thrombosis as an early warning sign of poor outcome.

The study has implications and limitations. The fact that a large number of Covid-19 patients experience arterial thrombosis casts doubts over the use of anticoagulants alone to prevent thrombosis sequelae. The mechanism accounting for thrombosis in Covid-19 has not been clarified yet. Hypoalbuminemia may be a factor favoring thrombosis but an interventional trial with albumin supplementation is needed to establish a cause-effect relationship. Furthermore, we have no information regarding the *quo ante* nutritional status, that may be an important element predisposing to low albumin levels. Finally, venous and arterial thrombosis may not share the same pathogenetic mechanisms, they could reflect different evolving conditions during hospitalization and could be associated with a different risk of poor prognosis. For example, acute limb or cerebral ischemic events could be related to a underlying new-onset atrial fibrillation, that is a common during pneumonia [25, 26]. Further studies will be necessary to understand the potential different implication of the different kinds of vascular complication and of their different timing in the disease course.

In conclusion, the present study provides further insights into the clinical picture of Covid-19, which is characterized by an equipollent incidence of venous and arterial thrombosis. Patients with thrombosis were affected by more severe disease and showed laboratory features of systemic inflammation and hypercoagulability. Such ischemic episodes are an ominous sign of poor survival, thereby early identification and appropriate therapy of at risk Covid-19 patients could manage the thrombotic risk and ameliorate mortality.
